# How health systems build capacity for antimicrobial stewardship: eight pillars to success

**DOI:** 10.1017/ash.2025.10102

**Published:** 2025-09-18

**Authors:** Reese A. Cosimi, Florian Daragjati, Melinda Mackey, Steve VanHook, Mohamad Fakih

**Affiliations:** 1 Ascension, St. Louis, MO, USA; 2 Wayne State University School of Medicine, Detroit, MI, USA

## Abstract

**Background:**

Antimicrobial stewardship (AMS) is crucial for improving infectious disease outcomes and mitigating antimicrobial resistance. Healthcare systems provide an ideal setting for implementing comprehensive AMS, but face challenges related to scale and complexity.

**Methods:**

This report describes Ascension’s system-wide AMS program, a model built upon establishing program structures, standardized processes, and fostering empowerment and partnerships. The program is structured around eight key pillars: system-level governance, market-level support, data-driven decision-making, multidisciplinary collaboration, data tracking and reporting, targeted interventions, empowering frontline teams, and stakeholder partnerships.

**Results:**

The system’s AMS program has yielded benefits, including the development and dissemination of standardized guidelines, the support of data-driven decisions through robust analytics and performance dashboards, and the deployment of clinical decision support tools. Capacity building has been enhanced through multidisciplinary collaboration and empowering frontline teams. Data tracking and reporting allow for the monitoring of key metrics.

**Conclusion:**

Healthcare systems can build capacity and support sustainability to AMS programs through developing structures, standardized processes, and empowerment to achieve optimal outcomes.

## Introduction

Infectious diseases represent a leading cause of mortality worldwide. Antimicrobial stewardship (AMS) programs mitigate the threats posed by antimicrobial resistance. Interventions implemented by AMS programs have resulted in 30% reduction in deaths associated with multidrug-resistant organisms (MDROs) in hospitals from 2012 to 2017.^1^ Much of the progress was halted during the first year of the COVID-19 pandemic, with a rise of 15% in MDRO infection associated mortality. This stark reversal highlights the ongoing urgency to strengthen AMS programs, across the continuum of care.

Healthcare systems provide an opportunity to bolster AMS across a myriad of settings. Yet, building capacity presents unique challenges.^2–7^ The complexity and scale of these systems, encompassing diverse patient populations, care settings, and healthcare professionals, necessitate adaptable strategies. Balancing the need for dedicated AMS personnel and technology with the overall resource allocation of the system can be challenging. Additionally, fostering a culture of AMS across a large, geographically diverse workforce requires sustained effort and engagement. Finally, collecting and analyzing comprehensive data across a vast network of facilities requires robust data infrastructure.

**Figure 1.**
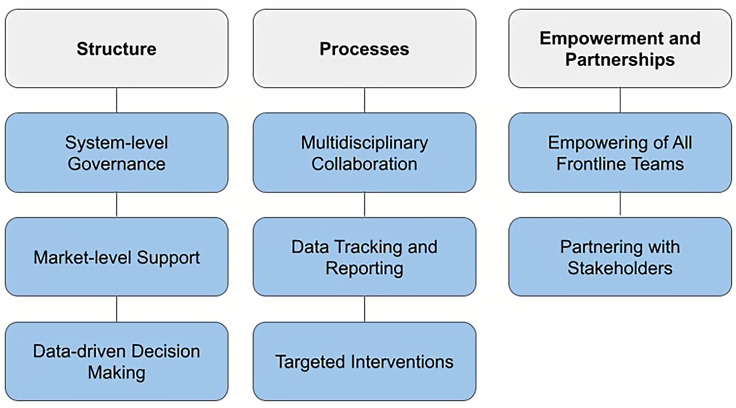
Eight pillars for an antimicrobial stewardship program in a large healthcare system.

We present Ascension, a system with 100+ hospitals and 2,600 outpatient sites, where we have established a system AMS program since 2015. Our system is a compilation of geographically distinct markets in 11 states. Here we address eight pillars that systems can implement to provide support for stewardship across their sites (Figure 1). We describe the structure that was built from system, to market, and local level to cascade stewardship efforts, provide concrete examples of processes to standardize and enhance outcomes, and emphasize real-time data access and accountability for sustainability.

### Building a program structure

In 2013, our evaluation of 85 Ascension hospitals showed variation in the structures and processes to support AMS, with half not having a formal stewardship program.^8^ Early goals included the standardization of processes for the effective management of common infections, improved efficiencies in care, reduction in antimicrobial resistant infections, and mitigation of the risk of *Clostridioides difficile* infections. In 2015, the program was expanded with the inclusion of a system AMS committee led by a system-level Infectious Diseases-trained pharmacist who reports as a director to the Chief Quality Officer and is supported by the committee co-chair Infectious Diseases physician. All markets are represented on the system committee by a pharmacist and physician champion who also lead AMS locally. The system AMS committee’s responsibility encompasses the standardization of all guidance to care related to stewardship, and provides system-level governance on program development and implementation. Three AMS subcommittees (Pediatrics, Ambulatory, and Microbiology) also support initiatives to standardize the work in their respective focus areas. Furthermore, all approved initiatives are then shared with the system Pharmacy and Therapeutics Committee, approved and disseminated as either expectations (required) or recommendations (strongly encouraged) across the system.

Market-level support, in the form of individual regional AMS committees, was also established and serve to cascade the guidance from the system committee and to provide support to the local teams. Local AMS programs were active in all hospitals by December 2015. In addition, we supported the formal education of 150 pharmacists through completing the Society of Infectious Diseases Pharmacists Stewardship Training Course. All the local hospital programs were aligned with the Center for Disease Control and Prevention’s (CDC) Core Elements for Hospital AMS Programs.^9^ A minimum of 1.0 full-time equivalent AMS-certified pharmacist support was instituted in each market, with some markets electing to exceed that baseline. This individual leads their peers in AMS in conjunction with an Infectious Diseases physician funded for a minimum of several hours a month dedicated to AMS. Supporting the function of the pharmacists to intervene and enhance the appropriate use of antimicrobials, we deployed a single clinical decision support (CDS) system in the vast majority of our hospitals with standardized actionable rules. Finally, we created dashboards that capture antimicrobial utilization and outcomes at the system, market, and hospital levels with benchmarking to identified targets. The dashboards also allow trending over time to evaluate progress. In 2017, Ascension gained national recognition for its achievement as a large healthcare system in prioritizing AMS, just two years after the inception of the systemwide standardized structure.^10–11^

By 2022, the system AMS program expanded to include all outpatient settings, governed by a national structure. A framework was developed and implemented in alignment with the CDC Core Elements for Outpatient AMS Programs.^12^ The framework included disease-specific guidelines, integrated point-of-care CDS, and provider education and engagement strategies. With the support of local physician and pharmacist champions and leaders, the framework was universally implemented with ongoing accountability reporting through monthly meetings with both the system- and market-level stakeholders. Furthermore, an outpatient antimicrobial prescribing dashboard was developed to support consistent auditing and feedback of individual clinician, clinic, and market prescribing rates with a focus on high-opportunity clinicians. Both the use of a CDS and dashboards across all settings support data-driven decision-making within the organization.

### Implementing key program processes

A cornerstone of our system’s program is the strong collaboration between various healthcare professionals. The system AMS committee is composed of infectious disease physicians and pharmacists, non-infectious disease physicians and pharmacists, nurses, microbiologists, infection preventionists, quality leaders, and clinical informaticists. The multidisciplinary representation in the market and local committees also mirrors the system level. This collaboration fosters knowledge sharing amongst thought leaders and key stakeholders, facilitates the implementation of evidence-based guidelines, and ensures effective rollout of standardized initiatives. With the goal of reducing clinical variation across the system, this multidisciplinary collaboration helps standardize practices across the continuum of care.

We use a robust data infrastructure to monitor and analyze antimicrobial use and resistance patterns, in conjunction with other infectious diseases-associated quality metrics (Table 1).^13–15^ Teams are able to access the data at the local, market, and system levels, and compare their performance to other Ascension sites or markets and the national level. The dashboards allow for all stakeholders to have real-time visibility into their progress and opportunities with numerous filters and drill-down functionalities. Outside of standard Antimicrobial Utilization and Resistance (AUR) reporting to the National Healthcare Safety Network, additional metrics are reported which include but are not limited to: antibiotic utilization, antibiotic resistance, process-based and outcomes-based metrics (Table 1).^16,17^ Performance is monitored and trended using the dashboards, and reports are shared regularly with leadership, clinicians, and other stakeholders, supporting informed decision-making and continuous program improvement. The dashboards are built to be flexible to updates and the development and refinement of new metrics. Further, clear lines of accountability are established for AMS activities, including defining roles and responsibilities for prescribers, pharmacists, infection preventionists, and microbiology. Nurses also have clear responsibilities such as obtaining appropriate cultures prior to starting antibiotics, identifying sepsis early, suggesting when to convert from intravenous to oral antibiotics, and documenting a detailed allergy history. Case Management as well is involved with clarifying antibiotic orders for discharge to optimize patient compliance. Leaders from each respective discipline conduct regular performance reviews and feedback mechanisms to monitor progress and identify areas for improvement with escalation pathways to the system-level champions and physician market leaders. The system AMS team also reports current AMS data on monthly Quality meetings with market leaders and conducts in-person market visits to strengthen relationships with the patient-facing teams and address opportunities and barriers.

**Table 1.** Standardized system-wide reported antimicrobial stewardship metrics

Metric	Definition	Data Source
Antimicrobial Utilization (Hospital - NHSN Reportable Units)	Days of Therapy per 1,000 Days Present Total Systemic Antibiotics and Individual Antibiotics Filters: System, Market, Hospital, Unit, Prescriber, Duration	NHSN AUR Report
Antimicrobial Utilization (Hospital - All Units)	Days of Therapy per 1,000 Length of Stay (Days) Total Systemic Antibiotics and Individual Antibiotics Filters: System, Market, Hospital, Unit, Prescriber	EHR Charge Report
Antimicrobial Utilization (Hospital - SAAR)	Observed / Expected (Days of Therapy per 1,000 Days Present) NHSN Defined Groupings Filters: System, Market, Hospital, Unit	NHSN SAAR Report
Antimicrobial Resistance (Hospital - Antibiogram)	Percent antimicrobial susceptibility of a specific microorganism Common microorganisms and All Antibiotics Filters: System, Market, Hospital	NHSN AUR Report
Antimicrobial Resistance (Hospital - SRIR)	Observed / Expected (Resistant infections) NHSN Defined Groupings Filters: System, Market, Hospital	NHSN AUR Report
Infectious Disease Outcomes (Hospital - Quality)^a^	Observed / Expected for Infectious Diseases (mortality, length-of-stay, readmissions, acute kidney-injury, etc.) Filters: System, Market, Hospital, Bloodstream infection by Pathogen	EHR Charge and Clinical Report
Infectious Disease Diagnostics (Hospital - Quality)	Diagnostic Test Utilization per 100 Encounters (Blood culture, Urinalysis, Urine Culture, CBC, CMP, Procalcitonin, Respiratory Viral Molecular Test, Rapid PCR, etc.) Filters: System, Market, Hospital	EHR Charge Report
Antimicrobial Utilization (Ambulatory)	Percent of Encounters Prescribed an Antibiotic^b^ Total Systemic Antibiotics and Individual Antibiotics Filters: System, Market, Clinic, Prescriber, Patient, Diagnosis	EHR Charge and Clinical Report

NHSN - National Health Safety Network; EHR - Electronic Health Record; AUR - Antimicrobial Use and Resistance Reporting; SAAR - Standardized Antimicrobial Administration Ratio^6^; SRIR - Standardized Resistant Infection Ratio^6^; CBC - Complete Blood Count; CMP - Complete Metabolic Panel; PCR - Polymerase Chain Reaction.

a Includes Medicare Severity Diagnosis Related Groups (MS-DRGs): 94, 95, 96, 152, 153, 177, 178, 179, 193, 194, 195, 288, 289, 290, 371, 372, 373, 485, 486, 487, 539, 540, 541, 548, 549, 550, 602, 603, 689, 690, 853, 854, 855, 856, 857, 858, 862, 863, 867, 868, 869, 870, 871, 872.

b Value-based purchasing criteria for antimicrobial utilization reporting applied where applicable^7^.

Not Listed: Infection prevention metrics reported by Ascension.

The system AMS program annually identifies a few high-impact priority goals. Specific evidence-based recommendations are developed to optimize antimicrobial prescribing and build upon the existing framework as a standardized effort.^9^ These targeted interventions include: prospective audit and feedback, antibiotic stewardship rounds, antibiotic preauthorization/restriction, development and implementation of local treatment guidelines (Table 2). Technology is also leveraged to hardwire standardized best practices, such as automatic stop dates for antibiotics, indication selection requirements, and cascade reporting for susceptibility results. The use of CDS tools (Table 3), disease state-specific order sets, and electronic-health record alerts further guide care across all settings. In the outpatient setting AMS is also supported by strategies such as watchful waiting, delayed prescribing, and payer-driven value-based reimbursement initiatives. This last component relates to a variety of measures that payers track and incorporate into scoring for systems to incentivize best practices, such as reducing antimicrobial prescribing for acute respiratory tract infections.^18^ These approaches have consistently maintained our Standardized Antimicrobial Administration Ratio (SAAR) for all antimicrobials below 1 with around 670, 140, and 86 Days of Therapy per 1,000 Days Present for all antimicrobials, broad-spectrum beta-lactams, and vancomycin, respectively.^19^ All the while keeping risk-adjusted mortality less than expected for infectious disease diagnoses, with observed/expected 0.746 and 0.674 for sepsis and pneumonia, respectively.^20^ Additional efforts to improve remdesivir prescribing for high-risk COVID-19 patients resulted in significantly less remdesivir use for Ascension compared to peers with consistently significantly less mortality than peer comparators.^21^ Antimicrobial prescribing in the ambulatory setting is also lower than payer-reported national averages for respiratory infections. Bronchitis being an example where percentage antimicrobial prescribing per encounter was reported as 48% for Ascension and payor comparators performing around 65%–80%.^22,23^

**Table 2.** Standardized system-wide AMS initiatives

Disease State Clinical Guidance	Appendicitis Beta-Lactam Allergy Assessment Bloodstream Infections Cellulitis *Clostridioides difficile* Treatment and Prevention COPD and Bronchitis Acute Exacerbation COVID-19 Treatment Guidelines Antifungal Treatment and Prophylaxis Meningitis - Bacterial Mpox Pneumonia Sepsis and Shock Treatment Surgical Antimicrobial Prophylaxis Surgical Irrigations Urinary Tract Infection/Pyelonephritis Ventilator-Associated Tracheitis (VAT) in Pediatrics Ambulatory Viral Respiratory Infections
Antimicrobial Dosing	Aminoglycosides Cefepime Daptomycin Meropenem Piperacillin-Tazobactam Vancomycin
Antimicrobial Restriction Criteria	Ceftaroline Dalbavancin/Oritavancin/Tedizolid Daptomycin Linezolid Telavancin Carbapenems Fluoroquinolones Ivermectin Ceftazidime-Avibactam Ceftolozane-Tazobactam Meropenem-Vaborbactam Eravacycline - Formulary (Tigecycline Non-Formulary) Colistin (Polymyxin E) and Polymyxin B
Antimicrobial Formulary	All Antimicrobials
Vaccine Guidance	Influenza Vaccine Recommendations Respiratory Syncytial Virus (RSV) Recommendations COVID-19 Vaccine Recommendations Pneumococcal Vaccine Recommendations Herpes Zoster (Shingles) Vaccine Hepatitis B Vaccine Recommendations
Diagnostic Stewardship	Blood Culture Collection and Testing Standardization *Clostridioides difficile* Testing Recommendations Gastrointestinal Panel Recommendations Influenza, SARS-CoV-2 and Respiratory Viral Panel Recommendations Microbial Cell-free DNA Test Polymerase Chain Reaction (PCR) Panels Methicillin-resistant *Staphylococcus aureus* (MRSA) Nasal PCR Screen to Rule Out MRSA Pneumonia Procalcitonin Recommendations Rapid Molecular Tests Urine Culture Collection, Testing and Resulting

**Table 3.** Standardized system-wide AMS clinical decision support pharmacy rules

Antimicrobial Stewardship (General)	Double Coverage for anaerobic organisms Double Coverage for atypical pathogens Double Coverage Beta-Lactam therapy Broad-Spectrum Beta-Lactam for>48 hours Bug / Drug Mismatch from Sterile Sites *Clostridioides difficile* on Inappropriate Gastrointestinal Medications Ceftriaxone at 48 to 72 hours for Pneumonia MRSA Screen on Anti-MRSA Agent MSSA Culture Streamlining Negative Influenza test on Oseltamivir Positive Cultures in 7 Days from Sterile Site Targeted Surveillance - Antifungals, Aztreonam, Cefiderocol, Ceftaroline, Ciprofloxacin, Colistimethate, Daptomycin, Eravacycline, Ertapenem, Fidaxomicin, Ganciclovir, Levofloxacin, Linezolid, Meropenem, New Gram Positive Agents, New Gram Negative Agents, Sulbactam / Durlobactam, Tigecycline, *Staphylococcus aureus* Bacteremia Care Bundle
Intravenous to Oral (Antimicrobials)	Azithromycin, Ciprofloxacin, Clindamycin, Doxycycline, Fluconazole, Levofloxacin, Linezolid, Metronidazole, Sulfamethoxazole / Trimethoprim, Voriconazole
Renal Dosing (Antimicrobials)	Acyclovir, Amphotericin B deoxycholate, Ampicillin, Ampicillin / Sulbactam, Aztreonam, Cefazolin, Cefepime, Cefiderocol, Cefotaxime, Cefoxitin, Ceftaroline, Ceftazidime, Ceftazidime / Avibactam, Ceftolozane / Tazobactam, Cefuroxime, Ciprofloxacin, Clarithromycin, Colistimethate, Daptomycin, Ertapenem, Fluconazole, Flucytosine, Imipenem / Cilastatin / Relebactam, Levofloxacin, Meropenem, Meropenem / Vaborbactam, Nitrofurantoin, Oseltamivir, Piperacillin / Tazobactam, Sulbactam / Durlobactam, Sulfamethoxazole / Trimethoprim, Valacyclovir, Valganciclovir
Pharmacokinetics (Antimicrobials)	Aminoglycosides Vancomycin
COVID-19 Therapeutics	Remdesivir, Baricitinib, Tocilizumab, Tofacitinib, Sarilumab, Nirmatrelvir / Ritonavir

### Supporting empowerment and partnerships

Our system AMS program supports empowering frontline teams with the knowledge, tools, and resources necessary to make informed decisions about antibiotic use. This is achieved through a multi-pronged approach. First, clinicians have access to CDS tools integrated directly into their electronic-health records and through external resources. This component is a major barrier to implementation. With more than eight electronic-health records across our acute care and outpatient settings, and limited information technology support, prioritization of high-impact enhancements is critical to avoid delays. Secondly, we offer educational and training programs on AMS principles, which are updated annually in collaboration with national clinical professional development teams. Third, the system AMS team engagement with individual markets supports the local AMS teams and brings accountability to the clinical leadership, reflected by their Chief Medical Officers. Finally, we promote patient engagement in care and shared-decision-making in the ambulatory space, which has been associated with improved patient satisfaction and outcomes in studies.^24,25^

The alignment of AMS efforts with quality and clinical initiatives considered as priorities for the system enhance their support and adoption. For example, “Recognize and Rescue”, a major initiative to improve and sustain acute care quality and safety across all our hospitals, provided a platform to increase the visibility of AMS as an integral component of improving infectious diseases outcomes.^19^ This framework which was implemented across all hospitals in 2022 identified high-risk conditions, defined best practices to promote safe and effective care for these conditions, and formalized a system-led accountability pathway which entails regular engagement of market teams including their executive leaders. While much of this work expands beyond infectious diseases, AMS remains a central focus. Sepsis management, in particular, was addressed as one of the priority high-risk conditions which benefits from a close involvement of the AMS team collaborating with the clinical teams. Using our data analytics dashboards, sites that are not meeting targets can be identified in real-time which allows for action to be taken by the market teams with the support and accountability structure funneling up to the system-level leaders to address barriers. Similarly, working closely with key hospitalwide stakeholders addressing diagnostic stewardship not only improves the use of antimicrobials, but optimizes reaching better diagnoses, and hence enhanced outcomes. Through alignment with systemwide priorities, AMS becomes more visible and valued as an important element to enhance patient outcomes.

Our system AMS team also collaborates with external partners, such as public health agencies, academic institutions, and professional societies to contribute to broader efforts to improve antibiotic use. As an example, delegates represented our system in The Pew Charitable Trust initiative to improve AMS for healthcare systems.^26^ Similarly, Ascension was recognized for their commitment to AMS by The AMR Challenge, an effort led by the United States government involving 350 organizations across the globe.^27^ Ascension was also recently awarded a grant by the Patient-Centered Outcomes Research Institute to support the management of pediatric respiratory tract infections in more than 200 outpatient sites.^28^ Such opportunity provides engagement with other health systems, establishing a learning environment amongst the network of organizations simultaneously participating in this work.

## Conclusion

The long-term success of any AMS program hinges on a sustainable approach that incorporates a standardized structure and processes. We provide an example of a successful health systemwide AMS program based on the eight pillars described herein. Health systems can play a central role in supporting AMS by supporting program structures, championing reliable processes, providing actionable data for the stewardship teams, ensuring accountability at all levels, and promoting sustainability, strategically advocating stewardship as a priority. Through a standardized approach, health systems across the globe can streamline workload and in turn improve patient outcomes and mitigate the risk of resistant infections.
